# The association of C-reactive protein to albumin ratio wıth lichen planus

**DOI:** 10.1590/1806-9282.20241166

**Published:** 2025-03-31

**Authors:** Hatice Ataş, Tuğba Atak, Selda Pelin Kartal, Gamze Tas Aygar

**Affiliations:** 1 University of Health Sciences, Diskapi Yildirim Beyazit Training and Research Hospital, Department of Dermatology and Venereology – Ankara, Turkey.; 2 Ministry of Health Islahiye State Hospital, Dermatology Clinic – Gaziantep, Turkey.

**Keywords:** Lichen planus, C-reactive protein, Albumin, Inflammation, Biomarkers

## Abstract

**OBJECTIVE:**

In some diseases associated with inflammation, the C-reactive protein-to-albumin ratio can be used as a biomarker of inflammation. Since lichen planus is a chronic inflammatory disease, our study aimed to investigate the relationship between C-reactive protein-to-albumin ratio and disease activity and whether it plays a role in determining disease prognosis, and compare them with those in subjects without lichen planus.

**METHODS:**

This is a case–control study. Demographic data, clinical features, and laboratory measures, including neutrophil, lymphocyte, eosinophil, platelet counts, neutrophile-to-lymphocyte ratio, erythrocyte sedimentation rate, C-reactive protein, albumin, and C-reactive protein-to-albumin ratio were statistically compared between patients with lichen planus (n=61) and controls (n=64).

**RESULTS:**

Albumin and erythrocyte sedimentation rate (p<0.001), C-reactive protein (p=0.017), C-reactive protein-to-albumin ratio (p=0.016), and neutrophile-to-lymphocyte ratio (p=0.02) were significantly higher in the patient group than in the control group. C-reactive protein-to-albumin ratio (p=0.003, OR 1.2), neutrophile-to-lymphocyte ratio (p=0.02, OR 1.4), and erythrocyte sedimentation rate (p=0.003, OR 1.1) were effective in differentiating patients from the healthy group. Erythrocyte sedimentation rate>4.5 mm/h, C-reactive protein-to-albumin ratio>0.6, and neutrophile-to-lymphocyte ratio>1 were useful in showing disease activity, of which C-reactive protein-to-albumin ratio has the best value with 92% sensitivity. C-reactive protein-to-albumin ratio has a sensitivity of 100% and is more effective and sensitive than other markers in distinguishing between mild and severe groups and between single and multiple involvement.

**CONCLUSION:**

Elevated C-reactive protein-to-albumin ratio levels may be considered a potential marker for lichen planus. It may be highly sensitive to follow-up of systemic inflammation and disease activity in patients with lichen planus. However, further prospective studies may confirm the association between C-reactive protein-to-albumin ratio and lichen planus.

## INTRODUCTION

Lichen planus (LP) is a chronic inflammatory disease of the skin, mucous membranes, hair, and nails. The pathogenesis of LP may be associated with infections and genetic and immune dysregulation, but it has not been completely understood^1^.

C-reactive protein to albumin ratio (CAR) was calculated as the ratio of serum CRP to serum albumin levels obtained from the biochemistry panel. It is considered to be a more informative measure of inflammation than either CRP or albumin alone^2^.

LP is characterized by elevated inflammation markers^3^. Accordingly, neutrophil-to-lymphocyte ratio (NLR) is a hemogram-derived inflammation marker that is reported to be associated with inflammatory conditions such as thyroid conditions, gastrointestinal diseases, diabetes mellitus, and cardiac conditions^4–6^. CAR has also been investigated in various malignancies, inflammatory diseases, and some dermatological diseases such as alopecia areata and psoriasis vulgaris^7–9^. As LP is an inflammatory disease, this study aimed to investigate its relationship with CAR, an inflammatory marker, and its correlation with disease activity. The assessment of this value in patients with LP will contribute to the literature. The aim of our study is to investigate the relationship between CAR and disease activity and whether it plays a role in determining disease prognosis.

## METHODS

This is a prospective single-center, matched case–control study. Patients diagnosed with LP clinically and histopathologically and who met the inclusion/exclusion criteria were included in the study. The blood samples were planned and demographic data were recorded. Biochemical data were recorded on the next follow-up. Patients who attended the dermatology clinic between January 2022 and December 2022 without a history of diabetes, hypertension, atherosclerotic heart disease, collagen tissue disease, additional inflammatory disease, and malignancy, and patients who did not use any drug that may affect serum CRP and albumin levels were included in the study. The control group was randomly recruited from patients presenting to our clinic with noninflammatory minor dermatoses such as nevi. Ethical approval was obtained (December 13, 2021, 126/01) and conducted in accordance with the Declaration of Helsinki guidelines. Informed consent was obtained.

Albumin, CRP, neutrophils, lymphocytes, sedimentation rate, platelets, and eosinophils were recorded in the blood analyses of the patients. Disease activity was calculated with the Lichen Planus Severity Index (LPSI) prepared by Kaur et al^10^. It was assumed that LPSI 1–3 was a mild disease (n=42) and LPSI 4–6 a severe disease (n=19). Patients were also divided into groups as single involvement (n=34) and multiple involvement (n=27).

Venipuncture was used to collect blood samples. The complete blood count was measured using the laser-based optical calorimetry method. An automated blood cell counter (Coulter LH 750 analyzer; Beckman Coulter, Galway, Ireland) was used. CAR was calculated as the ratio of serum CRP to serum albumin levels. The ratio of neutrophils to lymphocytes was used to estimate the NLR. A Westergren method-compatible automated erythrocyte sedimentation rate analyzer was used for erythrocyte sedimentation rate (ESR) (0–20 mm/h). CRP levels (<1 mg/dL) were measured using the immunoturbidimetric method.

### Statistical methods

Data were analyzed using SPSS-15. Kolmogorov-Smirnov test was used for normality test. Continuous variables were analyzed using the t-test or Mann-Whitney U test, and categorical variables were analyzed using the χ^2^ or Fisher's exact test. Multivariate logistic regression analysis was used to determine the influencing factors of LP. Receiver operating characteristic (ROC) analysis was used to identify an optimal cutoff value A p-value of <0.05 was considered to show a statistically significant result.

## RESULTS

The mean age (43.5±10.5 and 45.6±11.6, respectively) and gender distribution (29 females/32 males and 31 females/33 males, respectively) of the LP group and control group were similar. There was a family history of LP in 10 (16.4%) patients. The most common type of LP was the acute generalized type (n=25; 41%), followed by localized (n=16; 26.2%) and pigmented LP (n=7; 11.5%). There were also actinic in 3 (4.9%), actinic-erosive in 1 (1.6%), hypertrophic in 4 (6.6%), inverse in 1 (1.6%), lichenoid drug in 3 (4.9%), and localized pigmented in 1 (1.6%) patient/s. In the patient group, 17 (27.9%) had nail involvement and 16 (26.2%) had mucosal involvement. Most patients (n=18; 29.5%) had LPSI 2, followed by LPSI 1 (n=14; 23%) and LPSI 3 (n=10; 16.4%). There were nine (14.8%) patients with LPSI 4, six (9.8%) patients with LPSI 5, and four (6.6%) patients with LPSI 6. Albumin (p<0.001), CRP (p=0.017), ESR (p<0.001), CAR (p=0.016), and NLR (p=0.02) were significantly higher in the patient group than in the control group (Table 1).

**Table 1:** Clinical and laboratory results of subjects with lichen planus and healthy control.

Parameters	Patient (n=61)		Control (n=64)		p
	Mean	±SD	Mean	±SD	
Age (years)	43.5	±10.5	45.6	±11.6	0.29
Beginning age of disease (years)	41.9	±10.7			
Disease duration time (years)	1.5	±1.0			
Percentage of involvement area (%)	17.3	±16.1			
CRP (mg/dL)	19.1	±18.4	8.4	±7.3	0.017
Albumin (mg/dL)	3.7	±0.7	4.1	±0.4	<0.001
CRP/albumin ratio	6.3	±6.9	2.1	±1.8	0.016
ESR (mm/h)	10.9	±8.3	5.3	±4.7	<0.001
Neutrophil (x10^3^/μL)	4.9	±2.3	3.5	±1.5	0.59
Lymphocyte (x10^3^/μL)	2.0	±0.8	2.2	±0.8	0.15
NLR	3.1	±2.3	2.9	±1.5	0.02
Platelets (x10^3^/μL)	259	±50	255	±55	0.71
Eosinophil (x103/μL)	0.23	±0.33	0.2	±0.15	0.47

CRP: C-reactive protein; ESR: erythrocyte sedimentation rate; NLR: neutrophil/lymphocyte ratio; SD: standard deviation.

CAR, NLR, and ESR were effective in differentiating patients from the healthy group (Table 2).

**Table 2:** The effects of parameters of lichen planus in multivariate analysis.

Parameters	Univariate analysis			Multivariate analysis		
	p	OR	CI	p	OR	CI
Age (year)	0.29	0.98	0.95–1.01			
Gender (M/F)	0.92	0.96	0.48–1.9			
ESR (mm/h)	<0.001	1.2	1.1–1.2	0.003	1.1	1.1–1.2
CRP (mg/dL)	<0.001	1.1	1.1–1.2			
Albumin (mg/dL)	<0.001	0.3	0.1–0.6			
Neutrophil (x10^3^/μL)	0.16	1.1	0.9–1.1			
Lymphocyte (x10^3^/μL)	0.18	1.1	0.9–1.1			
Eosinophil (x10^3^/μL)	0.49	1.1	0.9–1.1			
Platelets (x10^3^/μL)	0.7	1.1	0.9–1.1			
CAR	<0.001	1.2	1.1–1.4	0.003	1.2	1.1–1.4
NLR	0.02	1.2	1.1–1.6	0.02	1.4	1.1–1.8

CI: confidence interval; CRP: C-reactive protein; ESR: erythrocyte sedimentation rate; NLR: neutrophil/lymphocyte ratio; OR: odds ratio; CAR: C-reactive protein-to-albumin ratio.

The median levels of the mild LP and severe LP groups were 1.2 (0.2–17.3) and 13.7 (range 1.8–24.2) (p<0.001) for CAR, 1.5 (range 1–6.3) and 3 (range 0.5–9.8) (p=0.002) for NLR, and 7.5 (1–30) and 12 (range 2–35) (p=0.03) for ESR. The median levels of the single involvement of LP and multiple involvement of LP groups were 1 (0.2–3.2) and 12.5 (range 0.9–24.4) (p<0.001) for CAR, 1.9 (range 1–9.4) and 2.8 (range 0.5–9.8) (p=0.18) for NLR, and 5.5 (1–30) and 12 (range 2–35) (p=0.007) for ESR.

The levels of ESR>4.5, CAR>0.6, and NLR>1 were useful in indicating disease activity. In addition, CAR (92%) and NLR (95%) sensitivity was similar but higher than ESR (75%). CAR has a sensitivity of 100% and is more effective and sensitive than other markers in distinguishing between mild and severe groups and between single and multiple involvement (Table 3).

**Table 3:** Receiver operating characteristic curve analysis of C-reactive protein/albumin, neutrophile/lymphocyte, and sedimentation.

Predicting	Parameters	AUC	p	CI	Cutoff	Sensitivity (%)	Specificity (%)
Presence of LP	CAR	0.63	0.02	0.52–0.73	>0.6	92	10
	NLR	0.61	0.04	0.5–0.7	>1	95	7
	ESR	0.74	<0.001	0.66–0.83	>4.5	75	60
Severity of LP	CAR	0.90	<0.001	0.83–0.97	>1.8	100	75
	NLR	0.73	0.06	0.60–0.86	>1.6	80	60
	ESR	0.72	0.03	0.53–0.81	>7.5	74	50
Extensivity of LP	CAR	0.97	<0.001	0.92–1	>0.9	100	40
	NLR	0.65	0.04	0.51–0.79	>1.6	80	45
	ESR	0.65	0.04	0.59–0.85	>7.5	78	60

AUC: area under the curve; CAR: C-reactive protein-to-albumin ratio; ESR: erythrocyte sedimentation rate; LP: lichen planus; NLR: neutrophil/lymphocyte ratio; CI: confidence interval.

## DISCUSSION

LP, which affects 0.1–1.27% of the population, has many types such as actinic, acute generalized, hypertrophic, and inverse. It can affect people of any age, with no gender or ethnic preference. The pathological mechanism of LP needs to be fully elucidated, as evidenced by the inadequacy of appropriate treatments. However, LP can be considered an inflammatory disease. CD4+ and CD8+ T cells that accumulate in the dermis are responsible for the apoptosis of basal epidermal cells through the secretion of various cytokines such as interleukin-2 and interferon-γ. These cytokines induce apoptosis of keratinocytes by cytotoxic CD8+ T cells^1,11,12^. In this study, positive inflammatory markers and ratios such as CAR, NLR, and ESR were found to be higher, and negative inflammatory protein albumin was found to be lower than controls. Also, serious disease and multiple involvement site levels gave the same high results. These results can support the inflammation in LP.

LP is known to be associated with dyslipidemia, and it has been shown that the risk of atherosclerosis may increase with the severity of inflammation^13^. Patients with LP were found to have higher metabolic and cardiovascular risk factors compared to controls, probably due to long-standing inflammation^14^. Therefore, it is important to monitor systemic inflammation with markers in LP. In our study, CRP, albumin, CAR, ESR, and NLR were significantly higher in the patient group than in the control group. Although CAR was statistically more significant in differentiating mild from severe disease, it was shown that NLR and ESR could also be used. The indicators of disease activity are ESR>4.5, CAR>0.6, and NLR>1. In differentiating between single and multiple involvement, CAR was again at the forefront, and it was shown that ESR could also be used significantly.

Tosun et al.^15^ were able to show an association between LP and the platelet/lymphocyte ratio or NLR, which are markers of inflammation, and NLR was an independent predictive factor for LP. The researchers believe that once the relationship between LP and NLR is understood, it could be used to determine the degree of inflammation in clinical follow-up. In our study, we found that NLR>1 might be associated with disease activity. In one study of patients with oral LP, serum CRP levels were found to be higher than in the control group, and in another study of the same type of patients, salivary CRP levels were found to be higher than in the control group^16,17^. Similar to these two studies, our study found high serum CRP levels in the patient group.

CAR and NLR have been investigated in many dermatological and inflammatory diseases. For alopecia areata, CAR value was found to be associated with disease activity with 87% sensitivity and 92% specificity. It can be used as an inflammatory marker in these patients^8^. Independent risk factors for disease activity of Behcet were disease duration (≤60 months), NLR (≥2), CRP (≥10 mg/L), and ESR (≥20 mm/H)^18^. In another study, CAR and NLR were positively associated with disease severity score in SJS/TEN (Stevens-Johnson Syndrome/Toxic Epidermal Necrolysis). NLR was also positively associated with CAR^19^. In the study of patients with psoriasis, the NLR was found to be associated with a high psoriasis area and severity index (PASI), and NLR has been proposed as an inflammatory marker in patients with psoriasis^20^. Kemeriz et al.^9^ showed that CAR was higher in the psoriasis group than in the control group and correlated strongly with PASI scores. In addition, CAR was more predictive of psoriasis severity than CRP, albumin, and ESR. When analyzed in patients with recurrent aphthous stomatitis, CAR values were higher in active lesions than in inactive lesions and higher in inactive lesions than in controls. The researchers concluded that CAR could predict the inflammation of these patients^21^.

Header et al.^22^ reported that CAR is important in showing the activity of ulcerative colitis. In another inflammatory disease, axial spondyloarthritis, CAR was found to be an independent predictive factor^23^.

Recently, CAR has been investigated as a prognostic marker in many cancers. An example of this can be seen in a study of patients with nasopharyngeal carcinoma. In this study, high CAR before treatment indicates poor diagnosis^24^. Preoperative CAR was also found to be a poor prognostic factor in resectable pancreatic cancer^25^.

LP is a chronic inflammatory disease. Increased CAR, ESR, and NLR in LP support the inflammation in our study. Increased inflammatory burden in some conditions such as severe and extensive diseases is reflected by serum CAR levels. In accordance with the literature as in the other diseases mentioned above, our results indicated the diagnostic value of CAR such as ESR and NLR. Especially in severe and extensive diseases, CAR might be a prognostic marker in LP. In addition to helping the diagnosis of LP, response to treatment and disease recurrence after treatment can be evaluated according to CAR levels in clinical use.

This study has some limitations, for example, many of the referenced articles discussed other dermatological, inflammatory, or medical diseases because there were not enough studies of LP on this topic. Furthermore, the study was conducted with a relatively small sample.

## CONCLUSION

As our findings and the existing literature indicate, CAR might be a marker with high sensitivity and a cutoff of 0.6 that can be used in relation to disease activity. It may play an important role in patient prognosis in the follow-up of systemic inflammation in patients with LP. However, further research is required to fully determine its impact on LP.
